# Severe immune mucositis and esophagitis in metastatic squamous carcinoma of the larynx associated with pembrolizumab

**DOI:** 10.1186/s40425-018-0332-z

**Published:** 2018-03-16

**Authors:** Fanny Zulay Acero Brand, Nicolas Suter, Jean-Philippe Adam, Bernard Faulques, Antonio Maietta, Denis Soulières, Normand Blais

**Affiliations:** 1 Faculty of Medicine, Department of Internal Medicine, Université de Montréal, Montréal, QC Canada; 2 Department of Pharmacy, Centre Hospitalier de l’Université de Montréal, Montréal, QC Canada; 3 Centre de Recherche du Centre Hospitalier de l’Université de Montréal, Montréal, QC Canada; 4 Department of Gastroenterology, Centre Hospitalier de l’Université de Montréal, Montréal, QC Canada; 5 Department of Pathology, Centre Hospitalier de l’Université de Montréal, Montréal, QC Canada; 6 Department of Medicine, Service of Hematology, Oncology and Blood Bank, Centre Hospitalier de l’Université de Montréal, 1051 rue Sanguinet, Montréal, QC H2X 0C1 Canada

**Keywords:** Pembrolizumab, Mucositis, Esophagitis, Immune-related adverse event

## Abstract

**Background:**

Pembrolizumab is an anti–*programmed death* 1 (PD-1) receptor monoclonal antibody that has shown activity as second line treatment for metastatic head and neck squamous cell carcinoma (HNSCC). Immune-related adverse events are now well described complications of PD-1 inhibitors and most organ sites have been shown to be potentially affected.

**Case presentation:**

We describe a 69-year old patient with a relapsed squamous cell carcinoma of the supraglottic larynx with lung metastasis after receiving adjuvant concurrent cisplatin and radiotherapy. This patient was treated with pembrolizumab and benefitted from therapy with major radiological improvement of disease. After 14 cycles of pembrolizumab 200 mg IV each 3 weeks, he experienced dysphagia that evolved to a grade 4 oral cavity and pharynx mucositis and esophagitis. Histologic analysis showed ulcerative esophagitis associated with granulation tissue. Pembrolizumab was discontinued and IV methylprednisolone 2 mg/kg/day was initiated. Two days later, the patient reported a 50% recovery in his symptoms which were completely resolved after 2 weeks. Methylprednisolone was switched to oral prednisone and a taper was planned over 8 weeks. During the fourth week of taper, the patient presented recurrence of grade 1 oral mucositis. Prednisone was increased 2 mg/kg/day for 2 weeks followed by slower tapering over a period of 5 months. Pembrolizumab was not reinitiated.

**Conclusion:**

This is the first described case of grade 4 immune mucositis and esophagitis associated with pembrolizumab. Because the use of pembrolizumab is increasing in oncology, pharmacists and physicians should be aware of this rare manifestation.

## Background

Pembrolizumab is an anti–*programmed death* 1 (PD-1) receptor monoclonal antibody that has received an approval by the Food and Drug Administration (FDA) for the treatment of metastatic melanoma, classical Hodgkin lymphoma (relapsed or refractory), unresectable or metastatic microsatellite instability-high cancer, PD-L1 expressing non-small cell lung cancer, advanced or metastatic urothelial carcinoma and metastatic or recurrent HNSCC after failure of a platinum containing regimen. This last indication was given based on promising results of two phase II studies [1, 2]. Well described immune related adverse events (irAEs) associated with pembrolizumab and other anti-PD-1/PD-L1 antagonists include dermatitis, hepatitis, pneumonitis and colitis. A case of isolated esophagitis associated with pembrolizumab [3] and a case of severe esophagitis and gastritis related to nivolumab have recently been reported [4]. Early recognition is the key to prompt management of these patients, which underscores the importance of describing atypical cases. We present such a patient who developed a grade 4 mucositis and esophagitis associated with the use of pembrolizumab. To our knowledge, this is the first severe immune mucositis associated with esophagitis published in the literature so far.

## Case presentation

A 69-year-old caucasian male with a history of cubital neuropathy and mild hypoacusia was diagnosed with T4N2M0 squamous cell carcinoma of the supraglottic larynx invading the thyroid and hyoid cartilages as well as the base of the tongue. He underwent total glosso-laryngectomy, tracheostomy and cervical lymphadenectomy. Treatment was pursued with adjuvant therapy including cisplatin 100 mg/m^2^ IV q 3 weeks for 3 cycles and concurrent radiotherapy (66Gy). Recurrence with multiple pulmonary nodular lesions occurred within six months after the end of adjuvant therapy. Three months later the patient initiated therapy with pembrolizumab 200 mg IV q 3 weeks within a clinical trial. During treatment, the patient developed an asymptomatic primary hypothyroidism (TSH = 6.87 mUI/L normal value between (0.30–5.50), T3 = 4.1 pmol/L (3.5–6.0) and T4 = 9.3 pmol/L (10.0–23.0)). Serum antithyroid peroxidase antibodies were not requested at the time of diagnosis. Because he had a normal baseline thyroid function, a diagnosis of hypothyroidism secondary to pembrolizumab was made and levothyroxine 50 mcg per day was initiated. The patient did not develop other immune related toxicity to pembrolizumab.

**Fig. 1:**
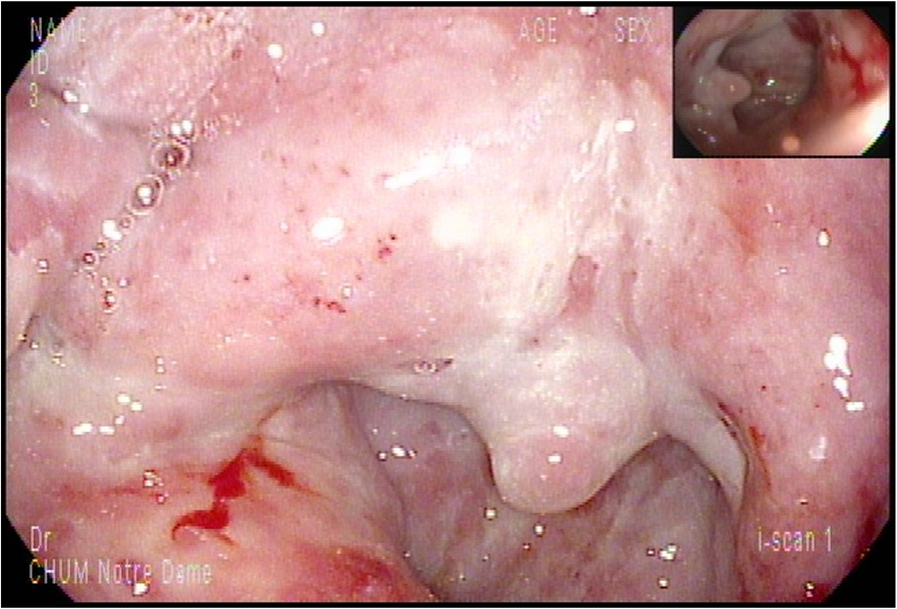
Mucositis: oropharyngeal ulcers with diffuse erythema

After 14 cycles, complete metastatic disease regression was documented in the right inferior pulmonary lobe, hilar, anterior mediastinal and pretracheal lymph nodes and in the right upper lobe micronodules and a left pulmonary micronodule was stable. At a follow-up appointment before cycle 15th, the patient experienced dysphagia. The physical examination demonstrated only small ulcers of the oral cavity. A treatment with sucralfate and magic mouthwash was given together with a 7-day course of valacyclovir based on a suspicion of herpetic involvement in a patient with a positive HSV-2 serology. Two weeks later, the patient was admitted to the hospital with a history of progressive dysphagia. He complained of oropharyngeal ulcers limiting swallowing that evolved to dysphagia to liquids and solids in the last 5 days as well as weight loss of 6 kg. The physical examination showed multiple painful oral cavity and oropharyngeal ulcers with diffuse erythema without any sign of oral thrush, any lacy white plaques (Wickham’s striae) or reticular white plaques (Fig. 1).

**Fig. 2:**
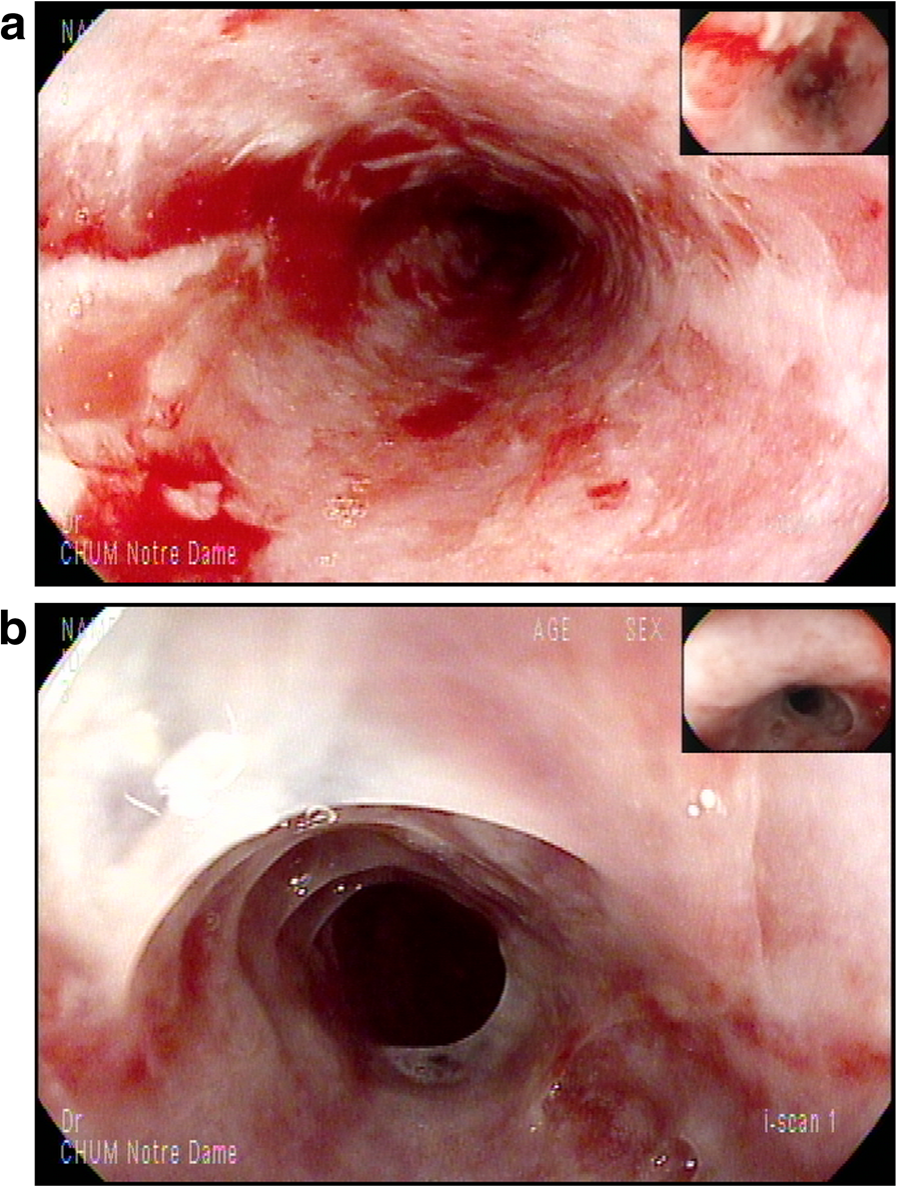
Gastroscopy. (**a**): Upper part of the esophagus showing severe circonferential esophagitis with superficial ulcerations, erythema and mild bleeding of the mucosa. (**b**): Distal part of the esophagus with aspect of dissequant esophagitis of the mucosa

**Fig. 3:**
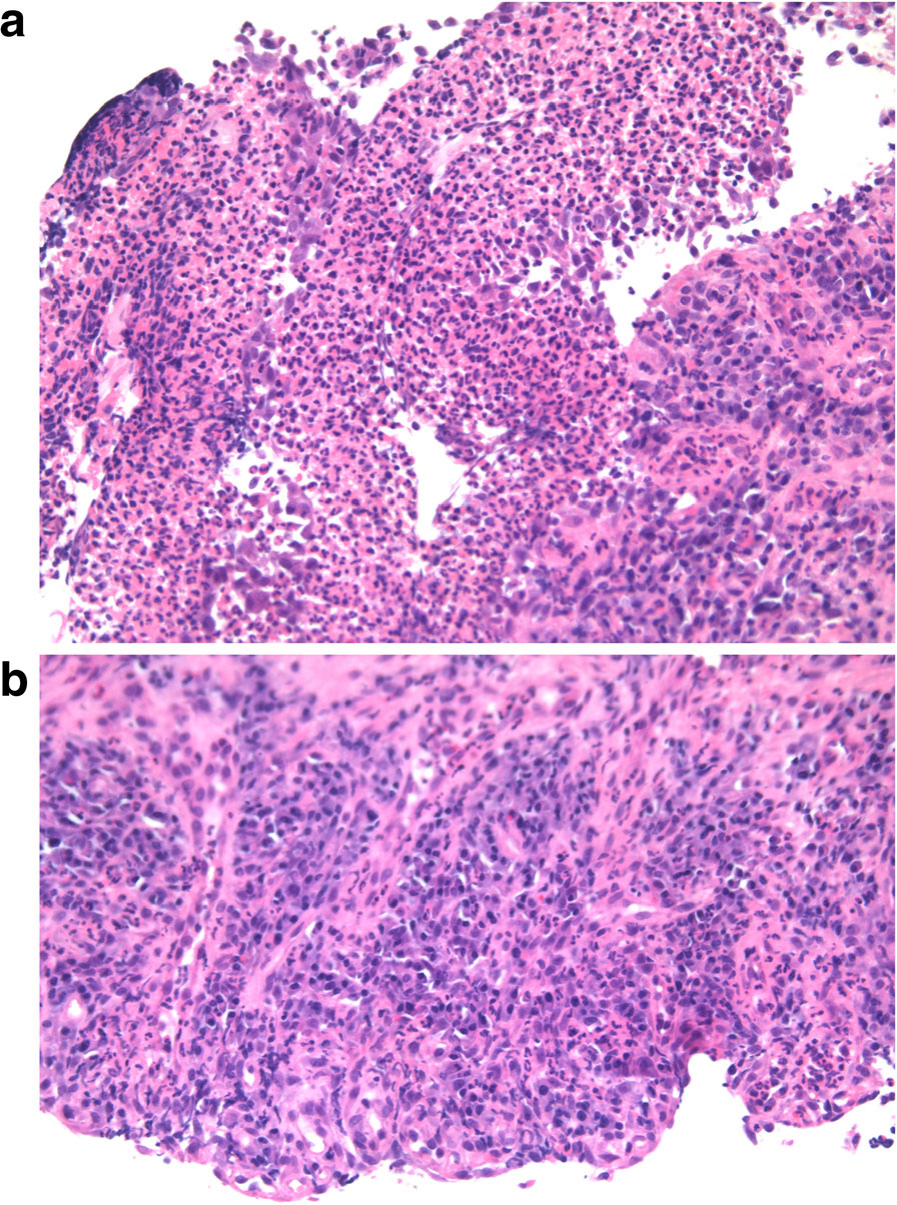
Histopathology (hematoxylin and eosin stain) of esophagus biopsy (× 200, panel **a** and × 400, panel **b**). Ulceration of the mucosa with granulation tissue

Initial blood tests at admission showed, hemoglobin 142 g/L, white cell count 7.7 × 10^9^/L (neutrophils 5.87 × 10^9^/L, lymphocytopenia 0.66 × 10^9^/L, eosinophils 0.05 × 10^9^/L) and platelets at 283 × 10^9^/L. All bacterial, viral and mycotic cultures were negative. IgG and IgM serology for CMV and EBV were also negative. Gastroscopy was performed and demonstrated severe ulcerating esophagitis with an appearance of esophagitis dissecans superficialis in the distal esophagus. Discrete hemorrhagic lesions were also present throughout the entire esophagus (Fig. 2). The biopsy showed ulcerated esophagitis associated with granulation tissue with absence of infectious features or malignant neoplasia (Fig. 3). Immunohistochemistry studies including, anti-CMV, anti-HSV 1 and 2 were negative.

Immune mucositis and esophagitis was considered the most likely clinical diagnosis and pembolizumab was temporarily discontinued. Intravenous methylprednisolone 2 mg/kg/day was initiated concurrently with the diagnostic evaluation and continued for 4 days. Marked improvement in symptoms was evident within 48 h. Treatment was thereafter switched to oral prednisone at 100 mg daily for 3 days with tapering planned over 2 months. Optimal analgesia, pantoprazole 40 mg twice daily and topical lidocaine were also given as supportive treatment. The patient reported complete relief of dysphagia at 2 weeks after hospital discharge. During the fourth week of taper, a grade 1 mucositis recurred at a dose of 20 mg of prednisone. Prednisone was re-escalated to 2 mg/kg/day. The patient tolerated a slow tapering of the prednisone thereafter without clinical evidence of recurrence. A follow up gastroscopy performed at 3 months showed incomplete mucosal healing. However, the patient remained with dysphagia that required a gastrostomy. Pembrolizumab was not restarted and the patient’s response was maintained for 8 months after pembrolizumab discontinuation, at which time a neck recurrence was documented. Prednisone was used over a period of 6 months.

## Discussion

Pembrolizumab is a humanized monoclonal antibody targeted against the PD-1 receptor. PD-1 is an inhibitory T-cell receptor that is engaged by two known ligands: PD-L1 and PD-L2 which are expressed within the tumour microenvironment and on tumor cells. Blockade of this receptor or its ligands restores immune recognition of tumoral neo-antigens found on tumor cells in several cancer types [5].

The expanded Keynote-012 study has shown that pembrolizumab has promising activity in metastatic or recurrent HNSCC with an overall response rate of 18% associated with a six-month progression-free survival and overall survival rates of 23% and 59%, respectively [2, 6]. Many responding patients are benefitting from prolonged remissions with ongoing treatment or even after treatment discontinuation. Our patient participated in an ongoing randomized trial and treated in the experimental pembrolizumab arm.

Adverse events of PD-1 checkpoint inhibitors are thought to arise following inhibition of auto-regulatory signals leading to autoimmune-like events. In Keynote-012, 62% of patients treated with pembrolizumab had at least one adverse event with 9% developing a grade 3 or 4 reaction [6]. IrAEs caused by anti-PD1 antibodyies have been well described involving mainly the gastrointestinal tract, liver and endocrine system [7]. Other rare irAEs including nephritis, meningitis, encephalitis, arthritis, myocarditis and pericarditis have also been described [7–9].

Oral mucositis caused immunotherapy is uncommon but seems more frequent with anti PD-1 inhibitors than with cytotoxic T-lymphocyte-associated antigen 4 (CTLA-4) inhibitors [10]. In the treatment of recurrent or metastatic HNSCC, the incidence of grade 1 and 2 mucositis is between 1 and 2% with nivolumab and pembrolizumab with no grade 3 or 4 events reported so far [1, 2, 11]. Isolated serious adverse events of upper GI involvement have rarely been reported with checkpoint inhibitors. Endoscopic and histopathological findings have not been well documented [12, 13]. One case of severe mucositis including ulcerative esophagitis at the fourth dose of pembrolizumab, without histological description and only one case of severe esophagitis associated with nivolumab in which pathological features are described were recently reported [3, 4].

Previous local radiotherapy does not seem to be a risk factor for mucositis as the majority of these patients have received concurrent chemoradiotherapy and the incidence of mucositis is the same as others studies in other tumor sites. In non-small-cell lung cancer, all grade stomatitis was report in 3% of patients with pembrolizumab 2–10 mg/kg, including one grade 3 event [14].

Differential diagnosis of mucosal toxicities includes dry mouth, oral candidiasis, lichen planus mucosae, gingivitis and sicca-like syndrome [9]. The incidence of dry mouth was reported in 6.5% of patients receiving nivolumab including one grade 3 toxicity [10]. Oral corticosteroids for management of other irAEs may be a risk factor for oral candidiasis. Infrequently, the development of oral lichenoid mucositis may be seen [9]. Two cases of lichen planus mucosae have been reported with both anti-PD1 agents. One grade 2 was reported with nivolumab after 43 weeks and one grade 3 was reported with pembrolizumab after 49 weeks [8]. In our patient, the clinical exam and biopsy results are not consistent with this latter diagnosis. Although the initial presentation was suggestive of herpes simplex mucositis, the lesions were not typical. The ulcers well not well circumscribed and did not present “volcano-like” margins. Moreover, the ulcers did not respond to antiviral treatment and the immunohistochemistry results were negative for HSV 1 and HSV 2. Of note, the area of the mucositis exceeded the area that was previously irradiated.

The Naranjo scale is a tool that can help evaluate the probability that the adverse drug reaction is actually due to one drug rather than other factors [15]. In our case, a score of seven was obtained with this tool, indicating a probable association between the administration of pembrolizumab and the severe immune event.

Endoscopically, the aspect of the lesions at the distal part of the esophagus were suggestive of esophagitis dissecans superficialis (EDS), a poorly described and rare desquamative disorder of the esophagus [16, 17]. Auto-immunity may thus be involved in such cases.

Management of irAEs is mainly symptomatic for mild events (grade 1–2), but will include high-dose steroids followed by slow tapering for more severe cases (grade 3–4) [10–19]. Most guidelines recommend suspending immunomodulatory agents in the presence of grade 3 (severe) adverse reactions until improvement to grade 0 or 1. Resumption of therapy is thereafter based on medical judgement, although the risk of recurrent grade 3 or 4 events have been well described [18]. In refractory cases, immunosuppressive agents such as mycophenylate mofetil, tacrolimus as well as TNF inhibitors [19] or anti-integrins may also be necessary [20]. But none of these options were necessary in this case of oral mucositis and esophagitis induced by pembrolizumab.

## Conclusion

Immunotherapy is an innovative part of cancer treatment. Despite promising safety results, serious adverse events are well reported. Close monitoring by well-trained physicians, pharmacists and nurses is an essential component to proper use of these agents. Mucositis is a rare irAE with pembrolizumab and mostly reported as grade 1 and 2. We describe a challenging case of pembrolizumab associated severe immune mucositis and esophagitis. Our patient showed complete clinical resolution of symptoms of grade 4 mucositis by 2-weeks of corticosteroids use, but experienced recurrence of grade 1 mucositis with tapering doses of steroids. For this reason, corticosteroids were re-escalated and pembrolizumab was suspended with careful follow-up scheduled. This adverse event was associated with a significant and ongoing tumoral response, even 6 months after drug interruption.

## Abbreviations

CMV: Cytomegalovirus
CTLA-4: Cytotoxic T-lymphocyte antigen-4
EBV: Epstein-Barr virus
EDS: Esophagitis dissecans superficialis
FDA: Food and drug administration
HNSCC: Head and neck squamous cell carcinoma
HSV-2: Herpes simplex virus type 2
irAEs: Immune related adverse events
PD-1: Programmed cell death protein-1
PD-L1: Programmed cell death ligand-1
PD-L2: Programmed cell death ligand-2
TNF: Tumor necrosis factor
